# Editorial: Ensuring food safety and quality in sustainable emerging production methods

**DOI:** 10.3389/fmicb.2025.1653076

**Published:** 2025-07-14

**Authors:** Alberto Garre, Lorena Martínez-Zamora, Ioana M. Bodea

**Affiliations:** ^1^Cold and Food Safety Engineering Group, Department of Agricultural Engineering, Universidad Politécnica de Cartagena, Cartagena, Murcia, Spain; ^2^Institute of Plant Biotechnology, Universidad Politécnica de Cartagena, Edificio I+D+I, Cartagena, Murcia, Spain; ^3^Postharvest and Refrigeration Group, Department of Agricultural Engineering, Universidad Politécnica de Cartagena, Cartagena, Murcia, Spain

**Keywords:** food safety, sustainability, antimicrobial resistance, food waste, artificial intelligence (AI), revalorization, real-time monitoring, public health

The Sustainable Development Goals (SDGs) reflect a global commitment to end poverty, protect the environment, and improve quality of life. Achieving these goals depends heavily on innovation in the food supply chain to ensure food security while minimizing environmental impact. New strategies in food production, distribution, and preservation (such as non-thermal methods, active packaging, AI-based systems, and waste revalorization) are emerging to support sustainable practices. However, these innovations may also introduce new safety challenges, including the risk of emerging or adapting pathogens. Continued scientific research is essential to evaluate and ensure the safety of food produced with these novel approaches. This Research Topic compiles eight recent studies highlighting advances in food safety and quality, focusing on the strengths and limitations of modern techniques for controlling foodborne microorganisms in the context of sustainability. Together, these contributions offer critical insights into how technological progress can support safer, more sustainable food systems without compromising public health.

Truchado et al. assessed four tertiary wastewater treatments: peracetic acid (PAA), PAA plus low-intensity UV-C, high-intensity UV-C, and ultrafiltration, in reducing extended-spectrum β-lactamase-producing *Escherichia coli* (ESBL-*E. coli*) and antimicrobial resistance genes (ARGs) in reclaimed water used for irrigation. Results showed that all technologies reduced ESBL-producing *E. coli* and resistance genes by at least ~1.5 and ~3 log cfu/100 mL, respectively, yet none achieved complete removal. Additionally, ultrafiltration performed best (~4-log ARG reduction), followed by high-intensity UV, while PAA-based treatments were less effective. The findings highlight the need of improved wastewater treatment strategies and stricter monitoring to mitigate in controlling antimicrobial resistance risks in water reuse systems intended for agricultural use. Champidou et al. evaluated the thermal inactivation of non-proteolytic type B *Clostridium botulinum* spores in a plant-based fish alternative to assess the effectiveness of heat treatments below standard safe harbor guidelines. Using inactivation treatments between 78 and 85°C, the time required for 6 log reduction in the plant-based food matrix was predicted at 1.26 min at 90°C. Results suggest that current guidelines for vacuum-packed chilled products, 90°C for 10 min, is approximately five times more than the time required for 6 log reduction of *C. botulinum*, indicating a substantial margin of safety.

Hanna Yumnam et al. identified 16 lactic acid bacteria (LAB) strains from traditional rice-based fermented products, highlighting their probiotic potential. The isolates showed strong tolerance to acidic and bile conditions, adhesion to intestinal cells, and no DNase or hemolytic activity were observed. Additionally, the identified LAB exhibited antimicrobial activity without undesired antibiotic resistance. While lacking certain enzymatic properties, their probiotic potential supports their use in functional foods and supplements, emphasizing the value of traditional fermented products as sources of beneficial bacteria. Additionally, Prieto et al. isolated different *Enterococcus faecium* and *E. lactis* from traditional Montenegrin cheeses and sausages, which were processed for whole-genome sequencing, revealing high genetic diversity and the presence of antimicrobial resistance, virulence, and bacteriocin genes. Whole-genome sequencing showed multiple plasmids and secondary metabolite genes, underscoring the potential risks associated with *E. faecium* and *E. lactis* highlighting the importance of ongoing monitoring.

Sulieman et al. explored the antifungal effect of *Pseudomonas* strains against *Phytophthora infestans*, a major threat to crops, with high impact on global agriculture and food security. Thus, nine *Pseudomonas* strains, with antifungal activity, were tested for their effectiveness. Results highlight the potential of these strains as biocontrol agents, emphasizing the complexity of microbial interactions and the importance of sustainable alternatives to chemical pesticides. This research supports advancing microbial-based disease management in agriculture, with recommendations for further field validation and ecological impact assessment. Furthermore, Qin et al. identified early warning biomarkers for detecting *Aspergillus flavus* and *A. niger* infection in maize kernels. By using GC-IMS analysis, 31 and 32 volatile organic compounds (VOCs) were detected in maize infected with MA and MB strains, respectively. Four characteristic VOCs: butan-2-one, ethyl acetate-D, benzaldehyde, and pentan-2-one, were identified as early markers, by appearing at 18 h of storage. These findings provided effective biomarker for the development of an early warning system for mold detection in stored maize.

Unis et al. described a sustainable method for the valorization of bread waste into high-quality protein and poly(3-hydroxybutyrate-co-3-hydroxyvalerate) PHBV biopolymer, by using the halophilic microorganism *Haloferax mediterranei*. By using enzymatically hydrolyzed bread waste, the process achieved notable biomass and biopolymer yields, with protein showing high digestibility and essential amino acid content. The protein quality, assessed *in-vitro*, indicated a high-quality protein with high digestibility, with a 91:9 mol% ratio of 3-hydroxybutyrate and 3-hydroxyvalerate. This approach offers a solution to food waste by generating valuable biomaterials and supporting circular bioeconomy goals. Liu and Li presented an integrated AI and blockchain (BCT) framework to enhance transparency, traceability, and early warning in green food supply chains. To achieve this goal, a two-part technical framework was developed. By using the ST-DBSCAN algorithm for anomaly detection and Hyperledger Fabric for blockchain-based traceability, the system achieves high accuracy, low latency, and strong data integrity. The model improved real-time risk monitoring and could support medium and small enterprises through green finance mechanisms, offering a practical, scalable and secure solution in food safety.

In conclusion, this Research Topic showcases how innovative technologies, and sustainable practices can work together to enhance food safety, sustainability, and resource efficiency. It addresses key challenges like antimicrobial resistance, food waste reuse, thermal processing, AI-based monitoring, probiotic use, and early detection of fungal contamination. While these advances are promising, they also present new risks, highlighting the need for continued research to safeguard public health.

Looking ahead, ensuring food safety and sustainability will require a flexible, forward-thinking approach. Research should focus on systems that can quickly identify and manage emerging risks as technologies evolve. Real-time monitoring, microbial detection, and predictive analytics will be crucial tools. At the same time, adopting environmentally friendly methods (upcycling food waste and using natural preservation) must be balanced with understanding their long-term impacts. Ongoing collaboration among scientists, industry, and policymakers will be vital to ensure responsible progress. The ultimate goal is to create food systems that are both innovative and safe, maintaining consumer trust while promoting environmental and public health.

